# Free propagation phase-contrast breast CT provides higher image quality than cone-beam breast-CT at low radiation doses: a feasibility study on human mastectomies

**DOI:** 10.1038/s41598-019-50075-6

**Published:** 2019-09-24

**Authors:** S. Pacilè, C. Dullin, P. Baran, M. Tonutti, C. Perske, U. Fischer, J. Albers, F. Arfelli, D. Dreossi, K. Pavlov, A. Maksimenko, S. C. Mayo, Y. I. Nesterets, S. Tavakoli Taba, S. Lewis, P. C. Brennan, T. E. Gureyev, G. Tromba, S. Wienbeck

**Affiliations:** 1 Elettra Sincrotrone Trieste S.C.p.A., Basovizza, Italy; 2 Department of Engineering and Architecture, University of Trieste, Trieste, Italy; 3 Institute for Diagnostic and Interventional Radiology, University Medical Center Goettingen, Goettingen, Germany; 4 Translational Molecular Imaging, Max-Plank-Institute for Experimental Medicine, Goettingen, Germany; 5 ARC Centre of Excellence in Advanced Molecular Imaging, School of Physics, The University of Melbourne, Parkville, Australia; 6 Department of Radiology, Academic Hospital of Trieste, Trieste, Italy; 7 Institute for Pathology, University Medical Center Goettingen, Goettingen, Germany; 8 Diagnostic Breast Center Goettingen, Goettingen, Germany; 9 Department of Physics, University of Trieste, Trieste, Italy; 10 School of Physical and Chemical Sciences, University of Canterbury, Christchurch, New Zealand; 11 Commonwealth Scientific and Industrial Research Organisation, Clayton, Australia; 12 School of Science and Technology, University of New England, Armidale, Australia; 13 School of Physics and Astronomy, Monash University, Clayton, Australia; 14 Australian Synchrotron, Clayton, Australia; 15 The University of Sydney, BREAST, Faculty of Health Sciences, Lidcombe, New South Wales Australia

**Keywords:** Cancer imaging, Biological physics, Imaging techniques

## Abstract

In this study we demonstrate the first direct comparison between synchrotron x-ray propagation-based CT (PB-CT) and cone-beam breast-CT (CB-CT) on human mastectomy specimens (N = 12) including different benign and malignant lesions. The image quality and diagnostic power of the obtained data sets were compared and judged by two independent expert radiologists. Two cases are presented in detail in this paper including a comparison with the corresponding histological evaluation. Results indicate that with PB-CT it is possible to increase the level of contrast-to-noise ratio (CNR) keeping the same level of dose used for the CB-CT or achieve the same level of CNR reached by CB-CT at a lower level of dose. In other words, PB-CT can achieve a higher diagnostic potential compared to the commercial breast-CT system while also delivering a considerably lower mean glandular dose. Therefore, we believe that PB-CT technique, if translated to a clinical setting, could have a significant impact in improving breast cancer diagnosis.

## Introduction

Breast cancer continues to be a major problem of public health with over 2 million new cases in 2018 world wide^1,2^. The main reason for the high mortality is late diagnosis. Thus, breast cancer screening programs have been established in several countries. In the framework of breast imaging, mammography is the only modality that has been shown to reduce breast cancer related mortality^1,3,4^. Nevertheless, it is limited by the fact that the recorded image represents the projection of a three-dimensional (3D) object onto a two-dimensional (2D) plane resulting in superimposition of structures. This already complicated situation gets worse in patients with dense breast tissue which results in stronger shadowing effects that can completely hide tumor tissue (leading to false negative diagnosis) or these dense regions might be falsely classified as tumor (false positives)^5–7^. X-ray based imaging techniques are using ionizing radiation which means that energy from the incident x-ray beam is transfered to the patient which leads to the known health risks, thus especially in cancer screening applications irradiation must be reduced as much as possible. Since the contrast in classical x-ray imaging is based on tissue specific differences in x-ray absorption, image quality and x-ray dose delivered to the patient are intrinsically coupled. Therefore, there is great need to develop alternative imaging strategies that deliver a reduced, or comparable, x-ray dose to patient combined with a reduction in false positives and/or false negatives diagnoses. Additionally, such methods should, ideally, remove the physical discomfort of breast compression required for mammography. Digital breast tomosythesis (DBT) - a technique that allows a limited stereotactic depiction of the breast - is an approach in that direction that has entered the clinics in recent years^8–10^. It has the potential to visually review thin breast section to unmask cancers obscured by normal tissue located above and/or below the lesion. However, it is not possible to focus on areas between two adjacent slices and the presence of micro-calcifications can, in some cases, cause artefacts in images^11,12^. A further development in that perspective is cone beam breast CT (CB-CT). CB-CT allows a true 3D visualization of the breast and has a proven higher ability to detect and characterize lesions within the breast^13–15^. CB-CT is a specially designed CT system in which detector and x-ray tube rotate around the breast and therefore only the interest region is irradiated. The technique is still based on x-ray attenuation principle as the classical CT, thus the spatial resolution and the signal-to-noise ratio that can be achieved are limited by the x-ray dose. Phase-contrast CT is an emerging imaging technique that exploits the wave nature of the incident x-ray beam, whereby providing images with a strongly increased contrast-to-noise ratio especially in soft-tissue applications compared to classical attenuation based x-ray imaging^16,17^. To date free propagation based PB-CT remains virtually limited to synchrotron light sources, but recent technological developments such as compact-light-sources point to possible clinical application in the future. The increased contrast-to-noise ratio of phase-contrast CT at comparable low x-ray doses would be ideally suited for breast cancer screening^18^. Therefore, we are working on phase contrast breast CT (PB-CT). Both modalities (PB-CT and CB-CT) have been studied and widely described in previous works^13–15,19–24^ but the comparison is hindered by the fact that experiments have been conducted on diverse samples. Here, we present the first direct comparison between CB-CT and PB-CT on a set of two human mastectomies.

## Methods

### Samples description

The study was performed in accordance with the Declaration of Helsinki and was approved by the institutional review board of University Medical Center Goettingen (ID 26/9/16). A written informed consent for excised breast material to be used for research purpose was obtained from all patients prior to their inclusion into the study. Two breast tissue specimens, which included an intraductal papillary carcinoma and an invasive ductal carcinoma with ductal carcinoma *in situ* (DCIS) respectively, have been imaged with both a dedicated breast CB-CT scanner (CB-CT 1000, Koning Corporation, USA) an University affiliated Medical Center in Goettingen (Germany) and with a PB-CT at the SYRMEP beamline of Elettra Synchrotron source (Italy). All samples, with thicknesses of approximately 3–4 cm, were formalin fixed and stored in sealed plastic bags.

### Absorption-based cone-beam breast-CT (CB-CT)

For the acquisition of absorption-based images, a Koning Breast-CT scanner was used. The system includes a horizontal CT gantry, an ergonomically designed exam table, an x-ray flat panel detector (PaxScan 4030 CB, Varian Medical System) and an x-ray tube with a 0.3 mm focal spot size (Rad 70, Varian Medical System)^13,25^. The physical pixel size of the detector was 194 $\mu$m, it has been used in 2 × 2 binning mode resulting in an effective pixel size of 273 $\mu$m (being the geometrical magnification equal to 1.42). The total acquisition time for each sample was 10 seconds for a $360$° rotation acquiring 300 angular projections. The acquisition parameters were: 49 kVp, 50 mA, 120 mAs and a mean glandular dose of 5.8 mGy (value given by the manufacturer).

### Phase-contrast breast-CT (PB-CT)

The PB-CT acquisitions were performed at SYRMEP beamline, where a clinical facility able to host patients is already designed. Here, the first program of clinical mammography with propagation-based phase-contrast was run^26^ and a new program for the implementation of PB-CT is ongoing^27^. Based on previous optimization works^23,28^ a sample-to-detector distance of 9.31 m was chosen, implying a beam cross-section at sample position of 220 mm (horizontal) × 3.5 mm (vertical, Gaussian shape, FWHM). A quasi-monochromatic x-ray beam with energies of either 32, 35 or 38 keV was used depending on the dimension of the samples. For the samples shown in this paper the selected energy was 32 keV. For all scans, 1800 projections were acquired, equally distributed over an angular range of $180$° with a total exposure time of 40 seconds. It has to be noticed that the exposure time is 4 times higher then the one of CB-CT acquisition just because of a technical limit of the rotating motor used for this PB-CT experiment. In a clinical set up a comparable exposure time can be reached. This means that PB-CT and CB-CT will be having the same level of patient movement artifacts.

To test if PB-CT can be applied for breast imaging technique in a clinical routine, different levels of radiation dose have been used, starting with approximately the same mean glandular dose (5 mGy) as delivered by the Koning system (allowing a comparison between the two techniques at a fixed level of dose), and also half of this dose. Calculation of the dose is based on a Monte Carlo simulation obtained using a numerical phantom that simulates a breast composed of 50 wt% glandular tissue and 50 wt% adipose tissue surrounded by adipose tissue (simulating the skin layer)^23,29,30^. The detector used was an XCounter photon counting detector (XC-FLITE FX2) with CdTe-CMOS technology and a physical pixel size of 100 $\mu$m. The geometrical magnification in this case was 1.405 resulting in an effective pixel size of 71.2 $\mu$m.

### Data processing

For PB-CT data processing, the X-TRACT software^31^ was used, which includes data pre-processing, phase retrieval and CT reconstruction. The pre-processing of projection images contained dark-current and flat-field correction and the application of a ring removal filter. Phase retrieval was performed using the TIE-Hom algorithm^32^. For CT reconstruction, the iterative filtered back-projection (iFBP) algorithm, also implemented in X-TRACT, was used. In order to obtain CT slices with the same thickness as for the CB-CT data, a 1D median filter on four reconstructed slices along their normal direction was applied. Due to the different acquisition geometries, the different positioning and possible structural changes of the samples over the time between the two experiments, a perfect image registration was not achievable, nonetheless qualitative conclusions could be drawn.

### Image comparison

The diagnostic value of the obtained images was examined by two independent expert radiologists, each with more than 30 years of experience in breast imaging. For each sample, the radiologists were asked to comment about features and details important for the diagnosis of the images obtained with both techniques and underline the strengths and weaknesses of each image. In particular they were asked to dedicate major attention to (1) the tumor margins, (2) the visibility of the spiculations, (3) the contrast of the lesion with respect to the surrounding healthy tissue, (4) the visibility of calcifications and (5) the overall noise in the image. In a second phase, radiologists were asked to focus only on the PB-CT images and to compare one image obtained at the initial dose (5 mGy) with one obtained at half of the initial dose (2.5 mGy). The goal of this further comparison was to understand if delivering half of the initial dose would affect previous diagnostic conclusions.

## Results

### Case 1 – Intraductal papillary carcinoma *in situ*

**Figure 1:**
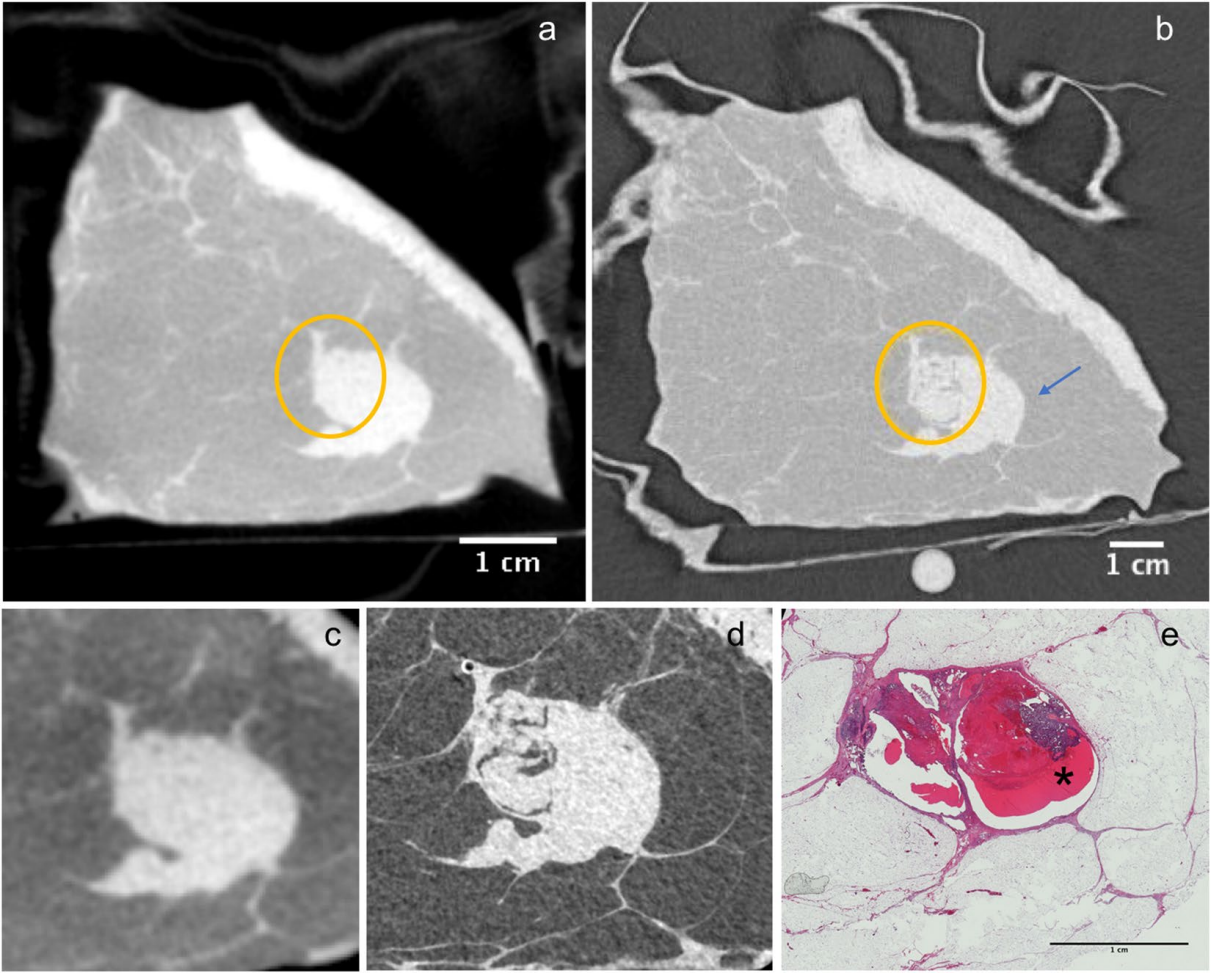
First case. Breast specimen from an 80-year old woman, including an intraductal papillary carcinoma *in situ*. (**a**) Image obtained with the dedicated CB-CT system in standard reconstruction mode. (**b**) Image obtained with PB-CT technique at 32 keV. The blue arrow indicates the part of the lesion with regular borders, the yellow circle highlights the infiltrating part. On the lower part of the figure a comparison between CB-CT image (**c**), PB-CT (**d**) and histology image (**e**) is shown. (**e**) Low power magnification (HE, 40×) showing a cyst with atypical papillary proliferation (*).

The first case in Fig. 1 includes an intraductal papillary carcinoma *in situ*. In particular, Fig. 1a,b show side by side the images of the CB-CT system and the PB-CT system acquired at the same radiation dose, while Fig. 1c,d show a close-up of the cancerous area. One of the most important notable aspects that differentiate the two images is the character of the tumor margins. On the CB-CT images, a lack of sharpness did not allow the reader to decide whether tumor cells were infiltrating the surrounding healthy breast tissue or not (yellow circle Fig. 1a). The PB-CT images were better able to depict the tumor composition in the tumor center than the CB-CT system. On the PB-CT images, since the borders of the lesion were sharper, it was possible to state that the right margin was regular (blue arrow Fig. 1b) and there were no signs of infiltration. Also on the left part (yellow circle Fig. 1a,b), one could see that a lobulated area could be compatible with an expansive growth. Finally, inside the lesion there is a fatty component that is clearly distinguishable on the PB-CT image but cannot be visually differentiated in the CB-CT system. Findings are verified by histology (Fig. 1e).

**Figure 2:**
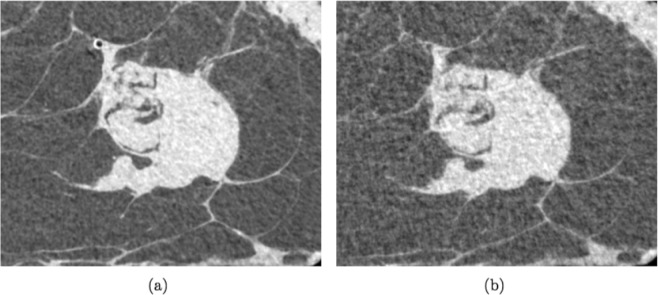
Close-up of first case breast specimen. Images obtained with PB-CT delivering a dose of 5 mGy (**a**) and 2.5 mGy (**b**). In (**b**) a more noisy background can be noticed together with a worse differentiation of the borders of the tumor lesion

For the second phase of the comparison of the first sample, even at a mean glandular dose of 2.5 mGy, PB-CT appeared to achieve better image quality than that of the CB-CT system. Figure 2 shows a close-up of the first sample including the area of the tumor. The low dose image is noisier and demonstrates a reduced delineation of the tumor lesion, however, it presents with similar radiological value as the higher dose image.

### Case 2 – Invasive ductal carcinoma with ductal carcinoma *in situ* (DCIS), intermediate grade

**Figure 3:**
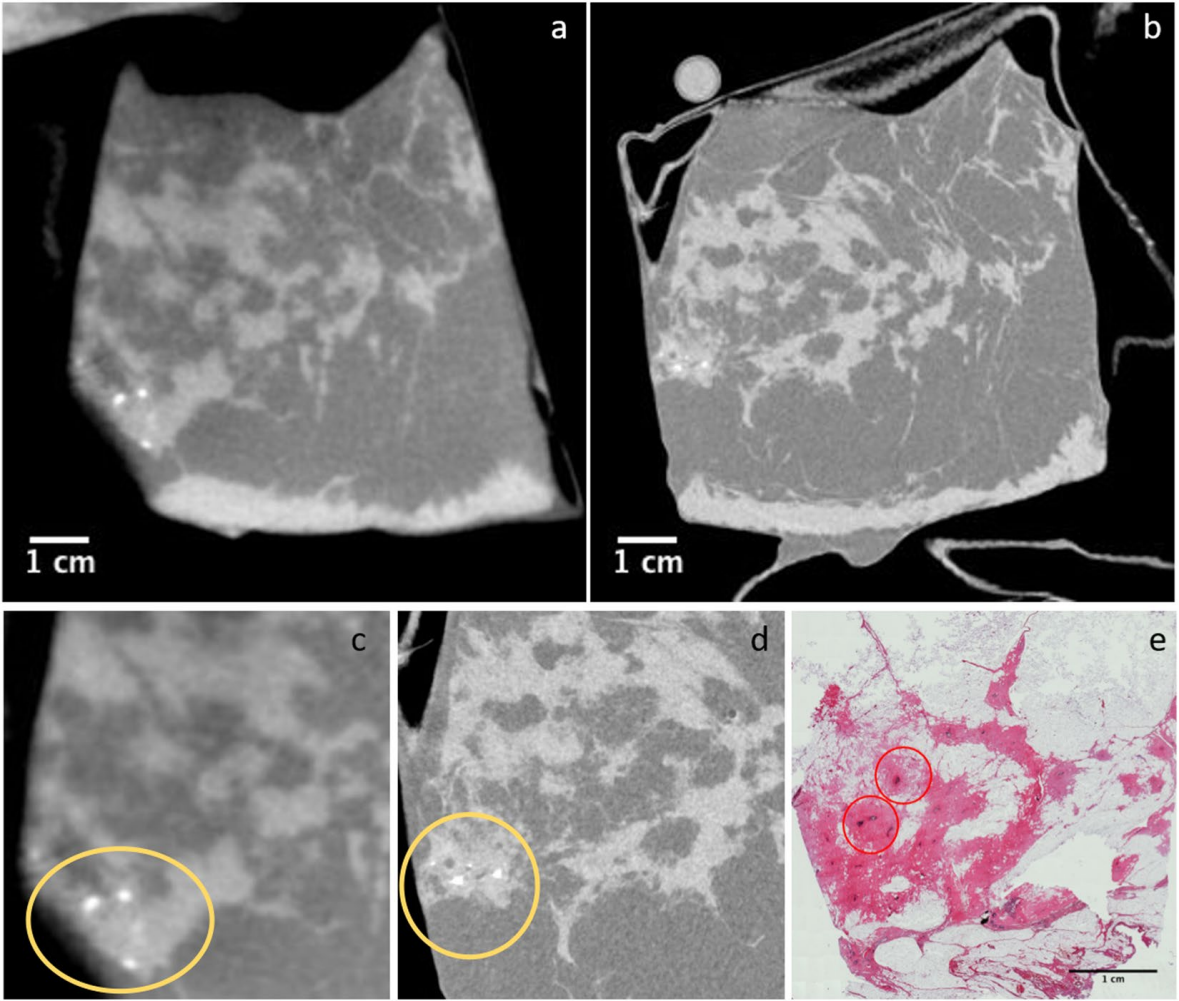
Second case. Breast specimen from a 62-year old woman, including an invasive ductal carcinoma with DCIS. (**a**) Image obtained with the dedicated CB-CT system. (**b**) Image obtained with PB-CT technique at 32 keV. Figures a and b show exactly the same specimen, however, a perfect image registration is not achievable due to the different acquisition geometries, the different positioning and the possible structural changes of the samples over the time between the two experiments. A close-up comparing CB-CT image with yellow circle indicating the micro-calcification area (**c**), SR image with yellow circle indicating the micro-calcification area (**d**) and histology (**e**) is shown. (**e**) Low power magnification (HE, 40×) showing a diffuse dense fibrosis with occasional small excretory ducts (red circles) with atypical ductal proliferation and micro-calcification.

The second case contained an invasive ductal carcinoma with a DCIS component. Figure 3a,b display the absorption-based CB-CT and the PB-CT, respectively. Again, the overall morphology of the lesion and the infiltration of the surrounding breast tissue were better visualized by the synchrotron radiation (SR) image. Margins were better defined, the morphology and distribution of the micro-calcifications were clearly detectable (brighter spots inside the tissue in Fig. 3c,d), and the higher contrast resolution allowed a better density differentiation. The dense fibrosis observed by histology 3e can also be seen in CT as more dense and brighter regions. It seems that the fibrotic tumor regions appear more heterogeneous than the normal fibrotic tissue within the breast, which due to the higher spatial resolution can more easily be seen in the PB-CT scan 3d. This again underlines the potential of the more dose efficient PB-CT in breast tumor imaging as it enables acquisitions at higher spatial resolution while maintaining the same dose like CB-CT.

## Discussion and Conclusions

Here we reported the first direct comparison between absorption-based CB-CT and synchrotron radiation PB-CT carried out scanning the same mastectomy specimens from two breast cancer patients. PB-CT and CB-CT were both performed with same x-ray dose of about 5 mGy (air kerma) which represented the scanning protocol with the lowest dose at the commercial CB-CT system. In accordance with previously obtained results^19–23^ we were able to realize a 2.5 times higher spatial resolution of about 100 $\mu$m in PB-CT compared to the pixel size of 273 $\mu$m in CB-CT. Improving the imaging resolution by using a detector with smaller pixels,normally comes at the expense of a higher dose, if the SNR is kept constant, or at lower SNR (per pixel), if the dose is kept constant. Our key result here, is that PC-CT was able to demonstrate a higher spatial resolution in the images at the same SNR and doses, as in Koning images. Since diagnosis often relies on the detection of subtle spiculations at the surface of the lesion, we found, in the first case of intraductal papillary carcinoma, a much better visualization of margins, lobes and spiculations, all critical to the diagnostic process. In the second case (the invasive ductal carcinoma) due to the nature of the particular cancer type, it is still possible to assess the malignant character of the lesion on the Koning CB-CT image, as the edges are very diffuse. However, because of the higher general sharpness of the image, the diagnosis would arguably be easier on the PB-CT image, as highlighted on Fig. 3d where a magnification of the area containing the micro-calcifications is displayed. In comparison with the commercial CB-CT scanner, in this feasibility study we have demonstrated that the gain in spatial resolution allows for a better discrimination of tumor spiculations and morphology and distribution of micro-calcifications. This last point is particularly important since clear visualization of micro-calcification (morphology and distribution) is used to differentiate between benign and malignant lesions, this being a common challenge of CB-CT imaging^33–35^. Moreover, the increased contrast resolution of PB-CT allows the analysis of density variations within the tumor region that we were not able to obtain in CB-CT. We believe that both these characteristics will contribute to a better breast cancer diagnosis using PB-CT technique whereby lowering the rate of false positive and/or false negative results. Additionally, an important aspect in breast cancer therapy is to evaluate the efficacy of a given treatment. Usually a successful treatment first modulates the blood supply of the tumor and thus changes the observed breast density^36–38^. However, assessing breast density changes with current mammography is difficult^39^ and the PB-CT would allow to address these effects being more sensitive to subtle density variations than conventional breast CT. To date, PB-CT has been limited to synchrotron light sources or micro-CT applications utilizing fine-focus x-ray tubes. A widespread use in clinical breast cancer diagnosis at the current time seems therefore unlikely. Nevertheless, the development of compact bright x-ray sources^40,41^ and novel liquid metal jet x-ray tubes^42^ already supports the use of phase-contrast CT imaging in biomedical research without synchrotron light sources and looks promising for future clinical applications. We therefore believe that, given this development, a clinical application of PB-CT can be expected in the near future.

Results of this feasibility study allow us to conclude that PB-CT can produce images with a higher quality compared to CB-CT systems, while delivering a lower level of radiation dose to the patient. However, in order to claim the highest diagnostic value of PB-CT technique, a quantitative and more specific blind evaluation performed by many radiologists on a larger number of specimens is foreseen. We also believe that phase-contrast tomography, if translated to a clinical setting, may improve breast cancer diagnosis accuracy. This imaging approach may for example be used to perform a high-resolution local area scans in a suspected breast tumor region previously localized in mammography or CB-CT and might therefore eliminate the need for biopsies in some cases.
